# Emphysematous Gastritis With Portal and Mesenteric Venous Gas Complicated by Septic Shock and Ileus: A Case Report

**DOI:** 10.7759/cureus.104164

**Published:** 2026-02-24

**Authors:** Dhiaalden Al Amri, Mustafa Al Hassani, Zaid Al Hassani, Aqeel Saleem, Muhammad Rab

**Affiliations:** 1 Internal Medicine, Sheikh Tahnoon Medical City, Al Ain, ARE; 2 General Practice, Sheikh Tahnoon Medical City, Al Ain, ARE; 3 Infectious Disease, Sheikh Tahnoon Medical City, Al Ain, ARE

**Keywords:** case report, computed tomography, emphysematous gastritis, ileus, portal venous gas, septic shock

## Abstract

Emphysematous gastritis (EG) is an uncommon, life-threatening cause of intramural gastric gas that may be accompanied by portal venous gas and shock.

An older adult with diabetes, vascular comorbidities, and chronic constipation presented with three days of worsening generalized abdominal pain following a home-administered enema and arrived profoundly hypotensive with metabolic acidosis and elevated lactate. Non-contrast computed tomography (CT) (limited by renal dysfunction) demonstrated gas in the posterior gastric wall consistent with emphysematous gastritis, with hepatic portal venous gas (HPVG) and mesenteric venous gas, and associated ileus without pneumoperitoneum.

The patient was managed conservatively in intensive care with hemodynamic support, bowel rest, nasogastric decompression, intravenous (IV) proton pump inhibitor therapy, broad-spectrum antimicrobials (with subsequent de-escalation), and parenteral nutrition, with clinical stabilization and discharge. Follow-up imaging and/or endoscopy was not completed as an inpatient due to family preference.

This case highlights the need for rapid imaging and multidisciplinary risk stratification when emphysematous gastritis is suspected in shock and illustrates the potential for recovery with carefully monitored non-operative management.

## Introduction

Emphysematous gastritis (EG) is a rare clinical entity characterized by the invasion of gas-forming organisms into the gastric mucosa [1]. The syndrome is typically recognized in critically ill patients, and diagnosis relies on the radiographic identification of air within the gastric wall that may extend into the portal venous system [2]. Reported mortality for EG has historically been high, reaching up to 60% in published case-based literature [3]. Predisposing factors described across reports include diabetes mellitus, immunosuppression, the ingestion of corrosive substances, alcohol misuse, and prior abdominal surgery, reflecting both systemic vulnerability and risks for mucosal injury [4].

A central diagnostic challenge is that intramural gastric gas (“gastric pneumatosis”) can also reflect gastric emphysema (GE), a condition that is often benign and managed supportively, whereas EG may necessitate aggressive resuscitation and, in selected cases, operative source control [5]. The prognostic divergence between EG and GE makes early clinical stratification essential, because similar symptoms and overlapping imaging patterns can mask markedly different underlying pathophysiology and risk [6].

Therapeutic decision-making for EG remains heterogeneous because robust prospective data are lacking and most guidance is derived from accumulated case experience [1]. Historically, early surgical exploration was frequently pursued; however, more recent reports describe the increasing use of medical management with surgery reserved for deterioration or complications [7]. In a Korean literature review focusing on EG with portal venous gas, the authors noted that aggressive surgical approaches have not consistently demonstrated prognostic benefit and may incur important postoperative complications, contributing to a trend toward avoiding immediate surgery in selected patients [8].

When EG is accompanied by hepatic portal venous gas (HPVG) or mesenteric venous gas, clinicians must simultaneously consider catastrophic ischemic or necrotic intra-abdominal pathology [9]. HPVG is considered an “ominous radiologic sign” in some contexts, but contemporary CT use has increased the recognition of benign etiologies, emphasizing that HPVG alone is not a surgical indication [10]. Accordingly, prognosis and management strategies are driven primarily by the underlying cause rather than by the radiographic finding itself [11].

This report describes a patient presenting with profound shock physiology and CT evidence of emphysematous gastritis with portal and mesenteric venous gas, treated successfully with intensive conservative therapy in conjunction with multidisciplinary surgical and gastroenterology evaluation.

## Case presentation

An 86-year-old retired man, living at home with family support, with a history of poorly controlled type 2 diabetes mellitus (HbA1c of 8.6% on admission), hypertension, hyperlipidemia, coronary artery disease, mildly reduced left ventricular systolic function, and chronic kidney disease stage 3, was brought by emergency medical services to the emergency department for severe worsening generalized abdominal pain. His regular medications included an antiplatelet agent, a statin, antihypertensive therapy, and glucose-lowering therapy. He denied nonsteroidal anti-inflammatory drug or corticosteroid use. He had no known drug allergies, and no prior abdominal surgery was reported. Functionally, he was awake and communicative at baseline, fed orally, and minimally mobile indoors.

He described the pain as “the worst abdominal pain I’ve had”: diffuse, constant, and progressively worsening over three days, with no clear relieving factors. The pain was accompanied by an inability to pass stool on a background of chronic constipation, for which he required regular laxatives, with bowel movements typically occurring every few days. A rectal enema was administered at home by a family member, after which his abdominal pain worsened markedly. He denied preceding nausea or vomiting, chest pain, palpitations, fever, respiratory symptoms, sick contacts, or recent travel.

On emergency medical service arrival, he was alert but in significant distress with an unrecordable blood pressure. He received 500 mL of 0.9% sodium chloride and ketamine pre-hospital. Approximately one month earlier, he had attended the emergency department with similar symptoms, declined admission, and left against medical advice.

On arrival to the resuscitation area, he remained profoundly hypotensive with an initially unreadable blood pressure. He was afebrile and maintaining an oxygen saturation of 91% on room air. The Glasgow Coma Scale score was 11/15, attributed to pre-hospital ketamine.

Examination demonstrated an ill-appearing patient with clinical features of poor peripheral perfusion, including cool extremities and delayed capillary refill, with dry mucous membranes. Cardiovascular examination revealed normal heart sounds without an audible murmur; jugular venous pressure was not elevated, and there was no peripheral edema. Chest examination revealed clear breath sounds with good air entry bilaterally.

Abdominal examination demonstrated marked distension with diffuse tenderness, without guarding, rigidity, or rebound tenderness, and no organomegaly was appreciated. Bowel sounds were reduced. Digital rectal examination did not reveal a palpable rectal mass; stool burden was present, and there was no gross rectal bleeding at the time of assessment.

Following initial resuscitation, blood pressure became recordable at 73/48 mmHg; respiratory rate was 19 breaths per minute, and oxygen saturation was 92% on room air. During ongoing resuscitation, he passed a large volume of melanotic stool and vomited partially digested food material streaked with blood. For circulatory support, he received repeated 500 mL boluses of 0.9% sodium chloride, hydrocortisone 100 mg, and 5% human albumin. Given persistent shock physiology, a norepinephrine infusion was commenced. Midodrine 10 mg was also administered as an adjunctive oral vasopressor but was not used as the primary treatment for shock.

Initial laboratory investigations are summarized in Table 1. The results showed acute kidney injury in chronic kidney disease with severe renal impairment, microcytic anemia, and venous acidemia with metabolic acidosis and elevated lactate, consistent with shock physiology. Inflammatory markers were not markedly elevated at presentation, and coagulation indices were within reference ranges.

**Table 1:** Baseline laboratory investigations at presentation

Tests	Results	Reference range (typical adult)
Sodium	140 mmol/L	135-145 mmol/L
Potassium	4.8 mmol/L	3.5-5.1 mmol/L
Chloride	109 mmol/L	98-107 mmol/L
Creatinine	291 µmol/L	62-106 µmol/L (male)
Estimated glomerular filtration rate	16 mL/minute/1.73 m²	≥60 mL/minute/1.73 m²
Lipase	83 IU/L	0-160 IU/L
Total protein	72 g/L	64-83 g/L
Globulin	28 g/L	20-35 g/L
Aspartate aminotransferase	11 IU/L	10-40 IU/L
Alanine aminotransferase	7 IU/L	7-56 IU/L
Alkaline phosphatase	196 IU/L	40-129 IU/L
Total bilirubin	7.3 µmol/L	3-20 µmol/L
Direct bilirubin	2.7 µmol/L	0-5 µmol/L
C-reactive protein	<1 mg/L	<5 mg/L
White blood cell count	9.3 × 10⁹/L	4.0-11.0 × 10⁹/L
Red blood cell count	5.12 × 10¹²/L	4.5-5.9 × 10¹²/L (male)
Hemoglobin	103 g/L	130-170 g/L (male)
Hematocrit	0.36 L/L	0.40-0.52 L/L (male)
Mean corpuscular volume	70.3 fL	80-100 fL
Mean corpuscular hemoglobin	20.1 pg	27-33 pg
Mean corpuscular hemoglobin concentration	286 g/L	320-360 g/L
Prothrombin time	11.8 seconds	11.0-13.5 seconds
International normalized ratio	0.96	0.8-1.2
Activated partial thromboplastin time	22.7 seconds	25-35 seconds
pH	7.20	7.31-7.41
Partial pressure of carbon dioxide	41.8 mmHg	41-51 mmHg
Partial pressure of oxygen	53.6 mmHg	30-50 mmHg
Bicarbonate (calculated)	16 mmol/L	22-26 mmol/L
Lactate (venous)	3.8 mmol/L	0.5-2.0 mmol/L

**Figure 1:**
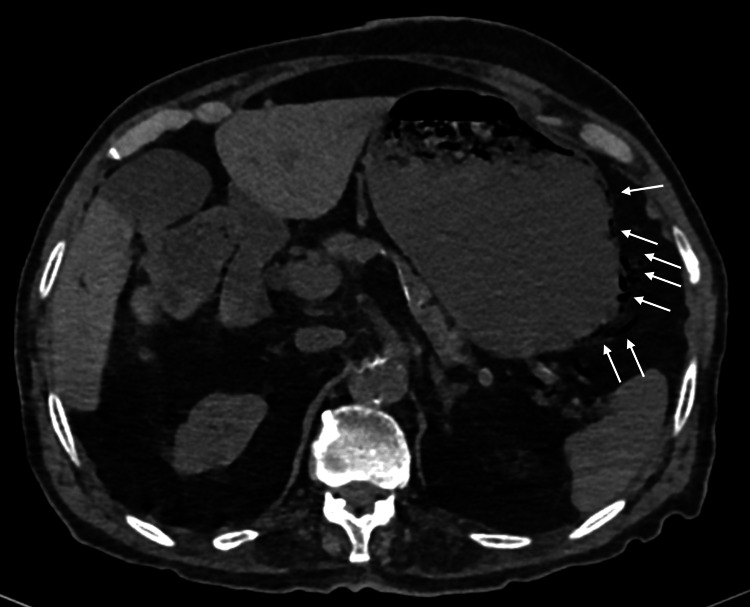
Non-contrast computed tomography (CT) demonstrating emphysematous gastritis with portal and mesenteric venous gas Non-contrast CT images show intramural gas within the posterior gastric wall, with hepatic portal venous gas and gas within mesenteric vessels. There is mild mural thickening at the splenic flexure/transverse colon with the dilatation of the colon and terminal ileum, consistent with ileus. No pneumoperitoneum, free intraperitoneal fluid, or drainable intra-abdominal collection is identified

Given the profound shock with severe abdominal pain and distension, urgent abdominal imaging was obtained to assess for perforation, ischemia, or other catastrophic intra-abdominal pathology. Non-contrast computed tomography (CT) (limited by renal dysfunction) demonstrated findings consistent with emphysematous gastritis with portomesenteric venous gas, along with features of ileus, and no radiologic evidence of perforation or drainable collection (Figure 1).

**Figure 2:**
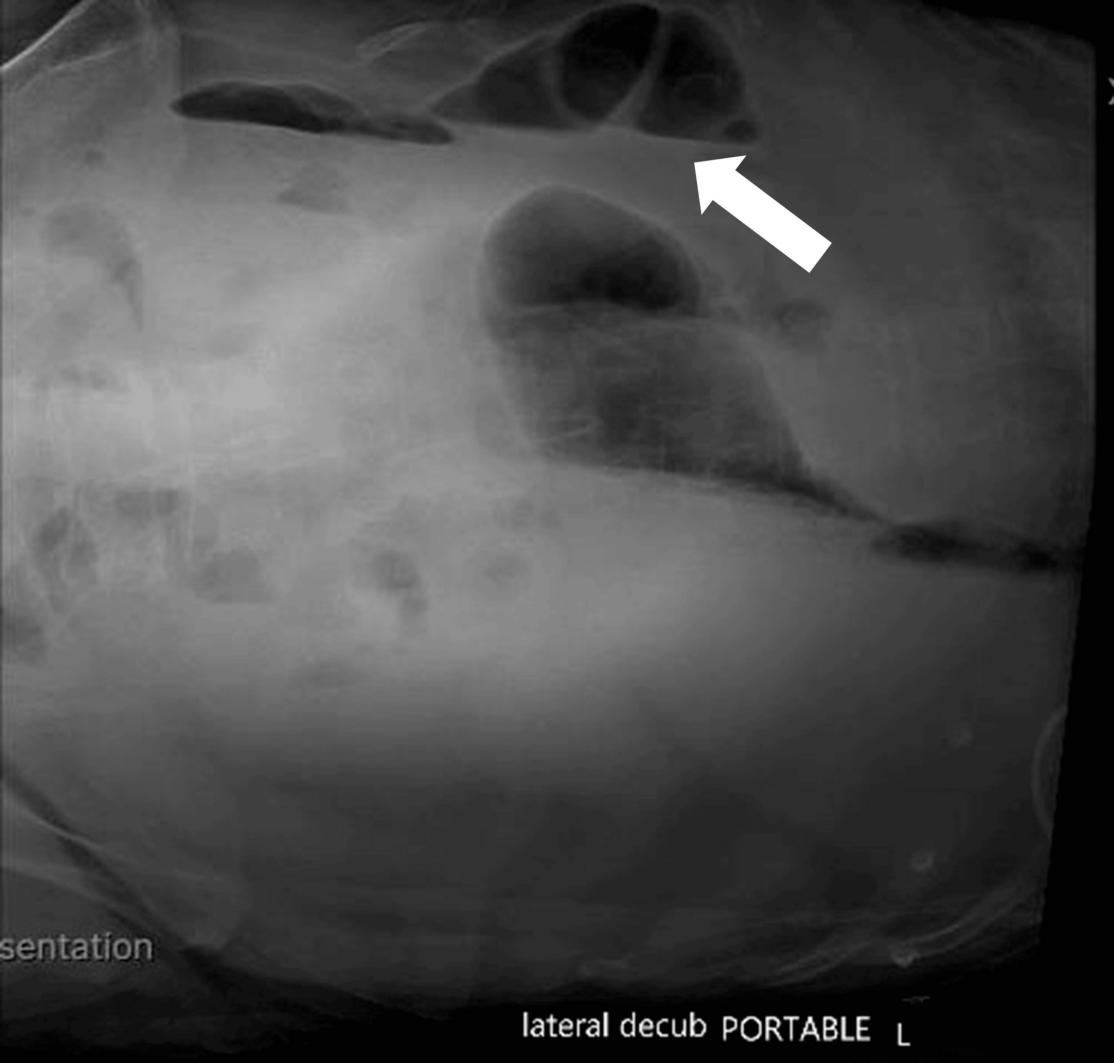
Abdominal radiograph demonstrating ileus with multiple air-fluid levels Abdominal radiograph series obtained on the day of presentation, including a lateral decubitus view, demonstrates diffuse gaseous bowel distension with multiple air-fluid levels, consistent with ileus

An abdominal radiograph series performed the same day showed gaseous bowel distension with multiple air-fluid levels (Figure 2).

Given the combination of gastric mural gas, portal venous gas, and shock physiology, gastrointestinal ischemia with superimposed infection was considered the leading mechanism, while mesenteric ischemia, perforated viscus, and gastrointestinal bleeding were also considered at presentation. However, the absence of peritoneal signs on examination, together with the lack of pneumoperitoneum or free fluid on imaging, reduced the likelihood of established perforation.

Gastroenterology and general surgery reviewed the patient and favored emphysematous gastritis with portal venous gas in the setting of shock. They recommended conservative management with strict fasting, nasogastric decompression, intravenous proton pump inhibitor therapy, and broad-spectrum antimicrobial therapy. Upper gastrointestinal endoscopy was deferred because of hemodynamic instability and concern for perforation through ischemic gastric mucosa, and surgery did not identify an immediate operative indication. The final diagnosis was emphysematous gastritis with hepatic portal venous gas and mesenteric venous gas presenting with septic shock physiology and ileus, complicated by metabolic acidosis and acute kidney injury in chronic kidney disease.

He was admitted to the intensive care unit (ICU) on day 1, requiring high-flow nasal oxygen (a fraction of inspired oxygen of 0.40 at 60 L/minute) and norepinephrine at approximately 0.1 µg/kg/minute. A nasogastric tube was placed for free drainage, and intravenous (IV) proton pump inhibitor therapy was continued (pantoprazole 40 mg IV twice daily, commenced on day 0-1 and continued until discharge). Empiric antimicrobial therapy with meropenem 1 g IV every 12 hours (day 1 to day 6; renal dose-adjusted) and linezolid 600 mg IV every 12 hours (day 1 to day 5) was initiated.

Arterial blood gas analysis showed partial improvement in acid-base status (pH of 7.27), and lactate decreased to 1.2 mmol/L. Over the next 24-48 hours, inflammatory markers rose (C-reactive protein of 22.9 mg/L and procalcitonin of 44.83 ng/mL) with leukocytosis and anemia (white cell count, 11.7 × 10⁹/L; hemoglobin, 86 g/L), as shown in Table 2. Urinalysis showed mild proteinuria and glycosuria without pyuria, and urine culture later returned negative.

**Table 2:** Laboratory investigations during early intensive care admission

Tests	Results	Reference range (typical adult)
Sodium	148 mmol/L	135-145 mmol/L
Potassium	4.5 mmol/L	3.5-5.1 mmol/L
Chloride	118 mmol/L	98-107 mmol/L
Creatinine	296.5 µmol/L	62-106 µmol/L (male)
Urea	31.80 mmol/L	2.5-7.8 mmol/L
C-reactive protein	22.9 mg/L	<5 mg/L
Procalcitonin	44.83 ng/mL	<0.05 ng/mL
White blood cell count	11.7 × 10⁹/L	4.0-11.0 × 10⁹/L
Red blood cell count	4.21 × 10¹²/L	4.5-5.9 × 10¹²/L (male)
Hemoglobin	86 g/L	130-170 g/L (male)
Hematocrit	0.30 L/L	0.40-0.52 L/L (male)
Mean corpuscular volume	71.3 fL	80-100 fL
Mean corpuscular hemoglobin	20.4 pg	27-33 pg
Mean corpuscular hemoglobin concentration	287 g/L	320-360 g/L
pH	7.27	7.35-7.45
Partial pressure of carbon dioxide	37.2 mmHg	35-45 mmHg
Partial pressure of oxygen	80.8 mmHg	80-100 mmHg
Bicarbonate	17 mmol/L	22-26 mmol/L
Lactate (arterial)	1.2 mmol/L	0.5-2.0 mmol/L

On day 2, anidulafungin was added due to severe illness in the context of diabetes (loading dose of 200 mg IV once and then 100 mg IV daily; day 2 to day 6), and total parenteral nutrition was commenced (day 2 to day 5) and then transitioned to peripheral parenteral nutrition (day 5 to day 7) before discontinuation as oral intake improved. Vasopressor requirements decreased as hemodynamics stabilized, and the Glasgow Coma Scale score improved to 13/15. During this phase, he developed significant electrolyte derangements, including hypernatremia and hypokalemia, which were corrected with supportive care, while renal function gradually improved (creatinine decreased from approximately 296 µmol/L to approximately 204 µmol/L), as shown in Table 3.

**Table 3:** Laboratory investigations during intensive care course

Tests	Results	Reference range (typical adult)
Sodium	156 mmol/L	135-145 mmol/L
Potassium	3.3 mmol/L	3.5-5.1 mmol/L
Chloride	117 mmol/L	98-107 mmol/L
Creatinine	204.2 µmol/L	62-106 µmol/L (male)
Urea	23.30 mmol/L	2.5-7.8 mmol/L
Calcium (total)	1.82 mmol/L	2.15-2.55 mmol/L
Corrected calcium	1.86 mmol/L	2.15-2.55 mmol/L
Procalcitonin	53.84 ng/mL	<0.05 ng/mL
C-reactive protein	253 mg/L	<5 mg/L
White blood cell count	16.2 × 10⁹/L	4.0-11.0 × 10⁹/L
Red blood cell count	4.83 × 10¹²/L	4.5-5.9 × 10¹²/L (male)
Hemoglobin	100 g/L	130-170 g/L (male)
Hematocrit	0.33 L/L	0.40-0.52 L/L (male)
Mean corpuscular volume	67.9 fL	80-100 fL
Mean corpuscular hemoglobin	20.7 pg	27-33 pg
Mean corpuscular hemoglobin concentration	305 g/L	320-360 g/L
Platelet count	200 × 10⁹/L	150-400 × 10⁹/L
Red cell distribution width	19.6%	11.5%-14.5%
Prothrombin time	13.4 seconds	11.0-13.5 seconds
International normalized ratio	1.22	0.8-1.2
Activated partial thromboplastin time	26.1 seconds	25-35 seconds
pH (venous)	7.44	7.35-7.45
Partial pressure of carbon dioxide (venous)	37.0 mmHg	35-45 mmHg
Partial pressure of oxygen (venous)	73.1 mmHg	80-100 mmHg
Bicarbonate (venous)	25 mmol/L	22-26 mmol/L
Lactate (arterial)	1.4 mmol/L	0.5-2.0 mmol/L

By day 3, respiratory support was weaned to low-flow nasal oxygen at 4 L/minute. Gastroenterology advised continuing parenteral nutrition and permitting cautious oral trials as tolerated, while endoscopy remained deferred due to ongoing clinical risk. By day 4, mental status improved further (Glasgow Coma Scale score of 14/15) with urine output approximately 60-80 mL/hour, but he was still considered unfit for endoscopy. On day 5, linezolid was discontinued, nutrition was transitioned from total parenteral nutrition to peripheral parenteral nutrition, and the central venous catheter was removed. Blood cultures obtained during admission later returned negative, and no microbiological organism was isolated. On day 6, swallowing assessment allowed a pureed diet, and antimicrobial therapy was de-escalated to piperacillin-tazobactam under infectious disease guidance.

On day 7, he was transferred from the intensive care unit to the medical ward. Parenteral nutrition was discontinued as his oral intake improved with swallow rehabilitation, and laboratory parameters continued to trend favorably, including improved renal function and decreasing inflammatory markers. Repeat abdominal radiography showed the regression of the air-fluid levels with no evidence of perforation.

By days 10-11, he was tolerating dysphagia-appropriate oral intake and passing stool and urine adequately and remained hemodynamically stable without vasopressors or advanced respiratory support. Gastroenterology recommended ongoing proton pump inhibitor therapy and interval repeat cross-sectional imaging, with or without endoscopy, to document resolution and exclude complications such as stricture. Antimicrobial therapy was completed during admission, comprising meropenem (day 1 to day 6), linezolid (day 1 to day 5), and piperacillin-tazobactam (day 6 to day 9).

The family declined further inpatient imaging, electing for outpatient gastroenterology review approximately one week later. Dietetics and swallowing therapy recommended a diabetic dysphagia diet with thin liquids and pureed foods, supplemented with diabetes-appropriate oral nutritional feeds. No clear extra-abdominal source of sepsis was identified; urine and blood cultures were negative, and chest radiography did not demonstrate a convincing infective focus, supporting emphysematous gastritis as the most plausible source of septic shock.

The patient was discharged clinically stable with a clear outpatient plan. Intervention adherence and tolerability were assessed through inpatient medication administration records, hemodynamic and respiratory trends, daily clinical review, and serial laboratory monitoring (renal function, electrolytes, and inflammatory markers). Notable adverse and unanticipated events included blood-streaked emesis at presentation with subsequent anemia, significant electrolyte derangements during intensive care (hypernatremia and hypokalemia) requiring correction, and a transient hypotensive episode on day 7 following the administration of sedating psychotropic medication, which resolved after the temporary withholding of pregabalin and quetiapine. The patient was discharged clinically stable on day 12 of hospitalization with a clear outpatient plan. Planned interval reassessment with repeat imaging and/or endoscopy was not completed in the hospital because the family declined further inpatient investigations. Subsequent outpatient follow-up attendance was not documented, representing a limitation of this report.

## Discussion

This case describes emphysematous gastritis with portal and mesenteric venous gas presenting with profound shock physiology and ileus, followed by clinical recovery under non-operative multidisciplinary management.

Emphysematous gastritis is most frequently encountered in critically unwell patients, and diagnosis relies on imaging demonstrating intramural gastric air that may extend into the portal venous system [2,5]. The clinical syndrome often includes abdominal pain with systemic upset and can include upper gastrointestinal bleeding manifestations such as hematemesis [2,5]. In the present case, shock and metabolic acidosis at presentation aligned with the severe end of the described EG spectrum and created immediate concern for ischemic or necrotic gastrointestinal pathology.

Pathophysiologically, EG is attributed to invasive, gas-producing organisms that inflame the gastric wall and dissect gas through tissue planes [12]. The microbiology reported in the literature is heterogeneous: Clostridial species are frequently cited, but enterobacteria, staphylococci, and fungi have also been identified, supporting broad empiric antimicrobial coverage when EG is suspected [12]. In addition to infectious drivers, many reports highlight disrupted mucosal integrity as a prerequisite for invasion, most commonly described in association with caustic ingestion and alcohol misuse, and accompanied by septic shock features [13]. Although the patient denied caustic ingestion and alcohol misuse, he had diabetes and vascular comorbidity and presented with hypoperfusion, which could plausibly contribute to gastric ischemia and mucosal injury, providing a substrate for secondary infection; this proposed mechanism remains inferential in the absence of endoscopic or histopathologic confirmation.

A major interpretive issue in cases of intramural gastric air is differentiating EG from gastric emphysema, because management and prognosis differ [5,6]. In gastric pneumatosis, radiographic discrimination can be difficult, and treatment ranges from supportive care for GE to gastrectomy for EG in severe cases [5]. Comparative review data suggest that EG mortality is driven primarily by sepsis and its complications, whereas GE mortality reflects comorbid disease burden, reinforcing the need to integrate physiologic severity with imaging interpretation [14]. In the present case, profound shock, metabolic acidosis, lactate elevation, and portal/mesenteric venous gas favored EG over benign GE, supporting escalation to ICU-level support rather than isolated supportive observation.

Imaging was pivotal to triage. Prior literature emphasizes that EG diagnosis rests on the radiologic demonstration of gas within the gastric wall, and that prompt diagnosis and treatment are central to survivorship [15]. Non-contrast CT, obtained because of severe renal dysfunction, still delineated intramural gastric gas and extensive portomesenteric venous gas, permitting rapid multidisciplinary decision-making despite the absence of intravenous contrast enhancement.

The presence of hepatic portal venous gas substantially broadens the differential diagnosis and historically signaled catastrophic bowel ischemia, but contemporary experience recognizes variable etiologies and outcomes [9,10]. A cumulative review of adult HPVG cases demonstrated that overall mortality varies with etiology and highlighted bowel necrosis as a frequent underlying event, supporting ongoing vigilance for ischemia when HPVG appears in an acutely ill patient [11]. Consistent with this approach, clinicians in this case pursued urgent imaging to assess for perforation and ischemia and weighed operative risk against the absence of peritoneal signs and the lack of pneumoperitoneum on CT.

The management of EG has moved toward carefully selected conservative treatment in some scenarios. Broad-spectrum antibiotics and supportive measures are commonly emphasized as early interventions because EG carries high mortality [13]. A case-based review of conservative EG management describes recovery using gastric rest (nil by mouth), nasogastric decompression, intravenous antibiotics, and intravenous proton pump inhibitor therapy, illustrating core elements of non-operative care [16]. More broadly, emerging commentary notes that historical gastrectomy-first strategies have largely been replaced by conservative therapy, including broad-spectrum antimicrobials, gut rest, and parenteral nutrition, with improved outcomes [2]. This trajectory is consistent with a cohort-style review of published EG cases reporting reduced mortality in cases described after 2000 and the increased utilization of endoscopy, alongside more selective operative intervention [3]. In the present case, conservative management was selected after surgical and gastroenterology evaluation found no immediate operative indication, and the patient improved with ICU support, antimicrobial therapy (including later de-escalation), nutritional support, and monitoring for complications.

The role of endoscopy remains unsettled and must be individualized. In gastric pneumatosis, more generally, early endoscopy has been proposed as a useful tool to aid differentiation between GE and EG and guide management strategy [2]. Conversely, recent commentary indicates that the role of esophago-gastro-duodenoscopy in EG is not established and is unlikely to be required in every case, particularly when physiologic instability raises procedural risk [5]. In this patient, endoscopy was deferred because of hemodynamic instability and concern for perforation through potentially ischemic mucosa; this decision likely reduced iatrogenic risk but limited diagnostic confirmation and assessment for mucosal necrosis.

Three further limitations affect interpretation. First, no organism was identified because cultures were negative, and no endoscopic biopsy was obtained; therefore, the exact microbiological etiology and the necessity of adjunct antifungal therapy cannot be confirmed. Second, stool investigations were not performed during the acute resuscitation phase, as management was prioritized toward hemodynamic stabilization and urgent imaging-based assessment; this limits the assessment of potential enteric infectious contributors. Third, planned interval imaging and/or endoscopy was not completed inpatient, and outpatient follow-up was not documented because the patient was lost to follow-up after discharge, preventing the objective confirmation of radiologic resolution (including portomesenteric venous gas) and the detection of late complications such as stricture or contracture [2].

## Conclusions

Emphysematous gastritis should be suspected when intramural gastric gas occurs with systemic toxicity or shock, particularly when portal or mesenteric venous gas is present. Hepatic portal venous gas is not an automatic indication for surgery; treatment and prognosis are driven by the underlying pathology, but intestinal ischemia must be urgently excluded in unstable patients. Conservative management (gut rest, nasogastric decompression, intravenous proton pump inhibitor therapy, broad-spectrum antimicrobials, and close monitoring) can be successful in selected cases without peritonitis or perforation. Endoscopy may help risk-stratify gastric pneumatosis, but its role in emphysematous gastritis is not established and should be individualized to physiologic stability and perforation risk. The documentation of follow-up imaging and/or endoscopy is important to confirm resolution and detect late complications; the absence of follow-up limits the interpretability of outcomes.
